# Trenton Medical Association

**Published:** 1876-04

**Authors:** 


Trenton Medical Association.—Dr. Ribble reported a case of *concussion of the brain, with cure, from free catharsis,* in a young man, twenty-four years of age, who jumped from his wagon while his horses were running away, and struck his head violently against a telegraph-pole, producing concussion of the brain. The patient remained in an insensible condition some three or four days, and then slowly regained consciousness.

At this time he described his condition as if he were in a dream. Various remedies were tried to better his condition, such as the bromides, iodides, stimulants and depressants, and bleeding, but all failed to give relief. The doctor remarked that he himself had suffered from concussion produced from a fall from his horse while in the Army of the Potomac, and had not obtained complete relief from the injuries sustained until a sharp and severe attack of dysentery set in. Reasoning from analogy, and while casting about for a remedy for this patient, he had been induced to try the same treatment that nature had employed in his case. Accordingly, he put the patient upon fld. ext. rhubarb and bi-carb. soda, producing free catharsis, which speedily relieved him of all the brain symptoms.

Dr. Elmer reported a case of persistent *vomiting during incipient pregnancy, successfully treated by dilatation of the os uteri*, after failure of all other remedies.

Dr. Green reported a case of *bleeding from gums* after extraction of teeth. The hemorrhage was severe, and he plugged the cavity with persulph. iron, followed by pressure, which arrested the bleeding for a time, but hemorrhage returned. A hypodermic injection of ergotin was then given, and arrested the bleeding at once.

Dr. Warman reported a case of *pseudomembranous croup* in which all the ordinary popular remedies, such as emetics, inhalations of vapor of lime, and lime-water, and finally filling the room with steam, failed to give relief, when, as a *dernier ressort* (the parents refusing to have the operation of tracheotomy performed), inhalations of *bromide of ammonium*, ten grains to the ounce, were used with the atomizer, and immediate relief from the most alarming dyspnœa was obtained, and the patient made a complete recovery, with the exception of entire loss of voice.

Dr. Warman reported also two cases in which *jaborandi* had been used for severe *catarrhal attacks*.

In both cases forty-five grains of the leaves had been given in infusion, with the peculiar characteristic result of the remedy, viz., profuse diaphoresis and salivation, and in both cases the colds that had been contracted were speedily broken up.

Dr. Rogers reported a case of *singular brain-disturbance from exposure to extreme cold*, as follows : A carter, aged about fifty years, after prolonged exposure to a low temperature, suffered from symptoms resembling acute congestion of the brain. Dr. Rogers found the man comatose, breathing stertorous, pulse slow, pupils dilated, and the temperature below normal. Stimulants were administered freely, together with artificial warmth, but for three days there was little or no change in the patient's condition ; at the end of the time mentioned, wild delirium supervened, without appreciable fever. The patient remained in this condition for two or three days longer, when he grew rapidly better, and was soon well. Since that time the man has pursued his ordinary occupation, his health being quite as good as usual. The doctor remarked that Lieutenant Payer, of the Austrian Arctic Exploring Expedition, had reported cases occurring among his crew very similar to this, produced by the extreme Arctic cold. Many of the sailors became delirious, and would have wandered off and died but for the fact that they were cared for by their comrades.

Numerous instances of the kind have been observed by Arctic explorers, but it is very rare that such phenomena as the results of exposure to cold are met with in this latitude. —*Medical Times.*

